# Evaluation of the comorbidity burden in patients with ankylosing spondylitis using a large US administrative claims data set

**DOI:** 10.1007/s10067-018-4086-2

**Published:** 2018-04-10

**Authors:** Jessica A. Walsh, Xue Song, Gilwan Kim, Yujin Park

**Affiliations:** 1 Division of Rheumatology, University of Utah School of Medicine and Salt Lake City Veteran Affairs Medical Center, Salt Lake City, UT USA; 2 Truven Health Analytics, an IBM Company, 75 Binney Drive, Cambridge, MA 02142 USA; 3 Novartis Pharmaceuticals Corporation, East Hanover, NJ USA

**Keywords:** Ankylosing spondylitis, Claims database, Comorbidities, Matched controls

## Abstract

Comorbidities among US patients with ankylosing spondylitis (AS) are inadequately understood. This study compared the prevalence and incidence of comorbidities between patients with AS and matched controls using national claims databases. Adults enrolled in the MarketScan Commercial and Medicare databases with ≥ 1 inpatient or ≥ 2 non-rule-out outpatient diagnoses of AS between January 1, 2012 and December 31, 2014 were included. Patients had to have ≥ 1 AS diagnosis in 2013; the first AS diagnosis in 2013 was assigned as the index date. Control patients without AS were matched to AS patients on age, geographic region, index calendar year, and sex. Comorbidities were evaluated in AS patients and matched controls during the baseline and follow-up periods (before and after the index date, respectively). Hazard ratios of developing new comorbidities were estimated using Cox proportional hazard models adjusted for patients’ characteristics. A total of 6679 patients with AS were matched to 19,951 control patients. In addition to extra-articular manifestations of AS (inflammatory bowel disease [IBD], psoriasis, uveitis), a higher proportion of AS patients had asthma, cardiovascular disease, depression, dyslipidemia, gastrointestinal ulcers, malignancies, multiple sclerosis, osteoporosis, sleep apnea, and spinal fractures during the baseline period than matched controls. After AS diagnosis, a higher proportion of patients developed newly diagnosed cases of cardiovascular diseases, depression, osteoporosis, spinal fracture, IBD, psoriasis, and uveitis than matched controls. In this real-world, US claims-based study, patients with AS were shown to have significantly more comorbidities than matched controls.

## Introduction

Ankylosing spondylitis (AS) is a systemic inflammatory disorder affecting the axial skeleton, peripheral joints, entheses, eyes, skin, and intestines [1–5]. Affecting 0.1 to 1% of the US general population, AS occurs more frequently in men, with a higher prevalence in whites [6–8]. The age of onset is typically the late teens through around 40 years. Onset after 50 years of age is unusual; however, delays in diagnosis are known to occur by as much as 8 to 11 years in some individuals [9–11].

Patients with AS often have extra-articular manifestations of AS, such as inflammatory bowel disease (IBD; Crohn disease and ulcerative colitis), psoriasis, and uveitis. Several studies, mostly conducted outside the USA, have also shown that patients with AS are at a higher risk of developing hypertension, hyperlipidemia, diabetes, peptic ulcers, headaches, depression, cancer, osteoporosis, and other cardiovascular, pulmonary, renal, and neurological complications compared with the general population [12–24]. Currently, there are a limited number of studies examining comorbidities in patients with AS in the USA. As rates of comorbidities in the general population are known to differ between the USA and other countries (e.g., obesity, diabetes, smoking), it is unknown if these differences affect comorbidity risk between patients with AS in the USA and those from other countries. This study aimed to examine the real-world comorbidity burden in US patients with AS using large national healthcare claims data and to compare comorbidities in patients with AS with matched control patients without AS.

## Methods

### Study design and data sources

This retrospective, observational cohort study used medical and pharmacy claims from January 1, 2012 through June 30, 2015, from two large administrative claims databases, the MarketScan® Commercial Claims and Encounters (Commercial) database and the Medicare Supplemental (Medicare) database. These databases include complete longitudinal records of inpatient services, outpatient services, long-term care, and prescription drug claims for commercially insured and Medicare-eligible patients covered under a variety of health plans from patients’ current or former employers. All database records are deidentified in full compliance with US patient confidentiality requirements set forth in the Health Insurance Portability and Accountability Act. Because this study used only deidentified patient records and did not involve the collection, use, or transmittal of individually identifiable data, institutional review board approval to conduct this study was not necessary.

### Patient selection

This study included patients aged ≥ 18 years with ≥ 1 inpatient or ≥ 2 non-rule-out outpatient medical claims for AS (*International Classification of Diseases, Ninth Revision, Clinical Modification* [ICD-9-CM] diagnosis code 720.0) > 30 days apart but within ≤ 365 days of each other between January 1, 2012 and December 31, 2014. Non-rule-out claims were those not related to a diagnostic or rule-out procedure (e.g., laboratory, pathology, or radiology services). Patients with AS were required to have ≥ 1 diagnosis of AS during 2013; the index date was the date of the first observed AS diagnosis in 2013. The year 2013 was chosen because of the availability of medical and pharmacy claims data at the time of analysis. Continuous enrollment with medical and pharmacy benefits for at least 12 months before the index date (baseline period) and 12 months after the index date (follow-up period) was required.

Patients with AS were matched to control patients without AS (general population) at a ratio of up to 5:1 based on age, geographic location, index calendar year, and sex. These matched controls had the same inclusion requirements as the patients with AS except for the diagnosis of AS. Matched controls were assigned the same index dates of their matched patients with AS.

All patients had a variable length follow-up period of ≥ 12 months until the earliest occurrence of inpatient death, end of continuous enrollment, or end of the data availability (June 30, 2015).

### Study variables

Patient demographics, including age, sex, geographic region, type of health plan, and duration of follow-up, were recorded on the index date. Comorbidities were identified using diagnosis codes in medical claims, but claims for diagnostic or rule-out procedures (e.g., laboratory, pathology, or radiology services) were excluded to avoid incorrectly identifying patients as having a comorbidity based on the history of testing rather than the test results. The following comorbidities were recorded during the baseline and follow-up periods: cardiovascular conditions (angina, atherosclerosis, cerebrovascular disease/stroke, coronary artery disease, hypertension, myocardial infarction, peripheral vascular disease, venous thromboembolism), IBD (Crohn disease, ulcerative colitis), gastrointestinal ulcers (esophageal, gastric, duodenal, peptic, and gastrojejunal ulcers), malignant neoplasms (malignant solid tumors, hematologic malignancies, and neuroendocrine tumors), asthma, depression, diabetes mellitus, dyslipidemia, multiple sclerosis, osteoporosis, Parkinson disease, psoriasis, sleep apnea, spinal fracture, and uveitis. Deyo-Charlson Comorbidity Index scores were calculated based on the presence of comorbidities during the baseline period. The ICD-9-CM codes used for these conditions are available upon request.

A comorbidity was considered to be newly diagnosed if the diagnosis code for this comorbidity was observed in the follow-up period but not in the baseline period. The incidence rates of comorbidities per 100 patient-years (PY) were calculated for patients with AS and their matched controls during the follow-up period. The incidence rate was calculated as ([number of patients with specific comorbidity/total observation days]/365) × 100. The total number of observation days was accumulated from the index date to the date of the first observed comorbidity for patients who developed a new comorbidity and the entire follow-up period for patients who did not develop a new comorbidity. Patients with comorbidities recorded in the baseline period were excluded from the incidence rate calculation.

### Statistical analysis

Bivariate descriptive analyses were conducted on all study variables comparing patients with AS and matched controls. Categorical variables were presented as counts and percentages; continuous variables were summarized with means and standard deviations. When appropriate, the statistical significance of cohort differences in bivariate descriptive statistics used *χ*^2^ tests for categorical variables and *t* tests for differences in continuous variables. The threshold for statistical significance was set a priori to the *P* value of 0.05.

Hazard ratios (HRs) were estimated using Cox proportional hazard models to assess the difference in the risk of developing a comorbidity between patients with AS and matched controls, overall and stratified by age (< 45 years, ≥ 45 to < 65 years, and ≥ 65 years); HRs were also estimated to assess the difference in the risk of developing a comorbidity between male and female patients with AS. HRs were adjusted for patients’ demographic characteristics (age, sex, health plan type, urban/rural residence, region, index year) and baseline clinical characteristics (Deyo-Charlson comorbidity index, cardiovascular disease, gastrointestinal disorders, malignancies, metabolic syndrome, neurological or psychological conditions, respiratory disease, osteoporosis, uveitis). The threshold for statistical significance was set a priori at 0.05. All analyses were conducted using SAS version 9.4 (SAS Institute Inc.).

## Results

**Fig. 1:**
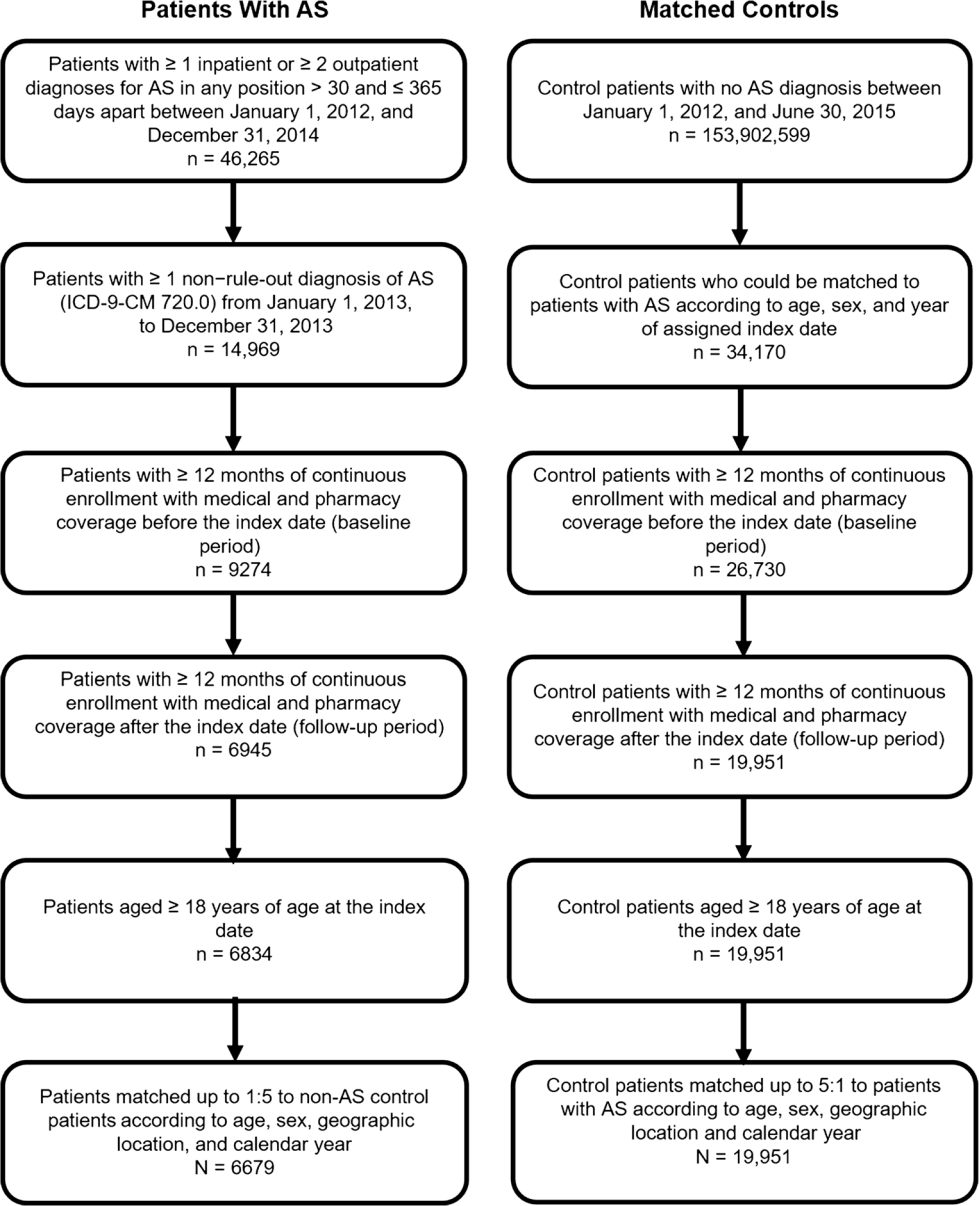
Selection of study cohorts. *AS*, ankylosing spondylitis. Non–rule-out claims were those not related to a diagnostic or rule-out procedure (e.g., laboratory, pathology, or radiology services). The index date was the date of the first *AS* diagnosis. Matched controls were assigned the same index date as their matched patient with *AS*

A total of 46,265 patients aged ≥ 18 years with ≥ 1 inpatient claim or ≥ 2 outpatient medical claims for AS > 30 days and ≤ 365 days apart were identified between January 1, 2012 and December 31, 2014, in the MarketScan Commercial and Medicare databases. A total of 6834 patients met the criteria for the AS cohort, and 6679 were matched with 19,951 patients without AS (Fig. 1). Patients with AS had a mean (SD) age of 50.8 (13.6) years, and 60.5% were men; matched controls had a mean age of 51.7 (13.4) years, and 60.8% were men. The SD length of follow-up in patients with AS and in matched controls was 739 (139) days and 740 (139) days, respectively (Table 1).

**Table 1:** Demographic and clinical characteristics of patients with AS and their matched controls

	Patients with AS	Matched controls	*P* value
	*N* = 6679	*N* = 19,951	
Age, years, mean (SD)	50.8 (13.6)	51.7 (13.4)	< 0.001
Age group, %			< 0.001
18–34 years	12.2	10.1	
35–44 years	19.4	18.6	
45–54 years	27.5	27.9	
55–64 years	28.2	29.7	
65+	12.8	13.7	
Male, %	60.5	60.8	0.647
Geographic region, %			< 0.001
Northeast	19.3	21.3	
North Central	21.1	21.9	
South	33.3	34.8	
West	25.2	21.1	
Unknown	1.1	0.9	
Health plan type, %			0.001
PPO	60.2	58.2	
HMO	11.9	11.9	
POS	7.9	8.1	
Comprehensive	7.6	8.0	
Other	12.4	13.8	
Days of follow-up, mean (SD)	739 (139)	740 (138)	0.858
Prior biologic exposure, %^a^	47.9	0.5	< 0.001

*AS*, ankylosing spondylitis; *HMO*, health maintenance organization; *POS*, point of service; *PPO*, preferred provider organization

^a^During baseline period

Patients with AS were shown to have a higher comorbidity burden than matched controls as evidenced by their higher Deyo-Charlson Comorbidity Index score in the baseline period (0.61 [1.15] vs 0.50 [1.14], *P* < 0.001). In addition, a significantly higher proportion of patients with AS were diagnosed with asthma, cardiovascular diseases, depression, dyslipidemia, gastrointestinal ulcers, malignancies, multiple sclerosis, osteoporosis, sleep apnea, spinal fracture, IBD, psoriasis, and uveitis than matched controls (Table 2). The proportion of patients with peripheral vascular disease, diabetes, and Parkinson disease was similar between patients with AS and matched controls.

**Table 2:** Baseline clinical characteristics and comorbidities in patients with AS and their matched controls

Comorbidities	Patients with AS *N* = 6679	Matched controls *N* = 19,951	*P* value
Deyo-Charlson Comorbidity Index, mean (SD)^a^	0.61 (1.15)	0.50 (1.14)	< 0.001
Comorbidities, *n* (%)			
Asthma	145 (2.2)	218 (1.1)	< 0.001
Cardiovascular disease	2295 (34.4)	5849 (29.3)	< 0.001
Angina	79 (1.2)	146 (0.7)	< 0.001
Atherosclerosis	482 (7.2)	1139 (5.7)	< 0.001
Cerebrovascular disease/stroke	110 (1.6)	260 (1.3)	0.038
Coronary artery disease	322 (4.8)	743 (3.7)	< 0.001
Hypertension	2053 (30.7)	5267 (26.4)	< 0.001
Myocardial infarction	84 (1.3)	177 (0.9)	0.009
Peripheral vascular disease	110 (1.6)	311 (1.6)	0.617
Venous thromboembolism	77 (1.2)	167 (0.8)	0.019
Depression	712 (10.7)	1152 (5.8)	< 0.001
Diabetes	656 (9.8)	2113 (10.6)	0.075
Dyslipidemia	1564 (23.4)	4439 (22.3)	0.048
Gastrointestinal ulcers	75 (1.1)	79 (0.4)	< 0.001
Malignancies	450 (6.7)	1094 (5.5)	< 0.001
Multiple sclerosis	31 (0.5)	51 (0.3)	0.008
Osteoporosis	259 (3.9)	191 (1.0)	< 0.001
Parkinson disease	17 (0.3)	43 (0.2)	0.561
Sleep apnea	585 (8.8)	1008 (5.1)	< 0.001
Spinal fracture	59 (0.9)	39 (0.2)	< 0.001
Extra-articular manifestations of AS, %			
Inflammatory bowel disease	439 (6.6)	128 (0.6)	< 0.001
Crohn disease	281 (4.2)	57 (0.3)	< 0.001
Ulcerative colitis	192 (2.9)	81 (0.4)	< 0.001
Psoriasis	138 (2.1)	171 (0.9)	< 0.001
Uveitis	654 (9.8)	45 (0.2)	< 0.001

*AS*, ankylosing spondylitis

^a^Deyo-Charlson Comorbidity Index ranges from 0 to 33

**Fig. 2:**
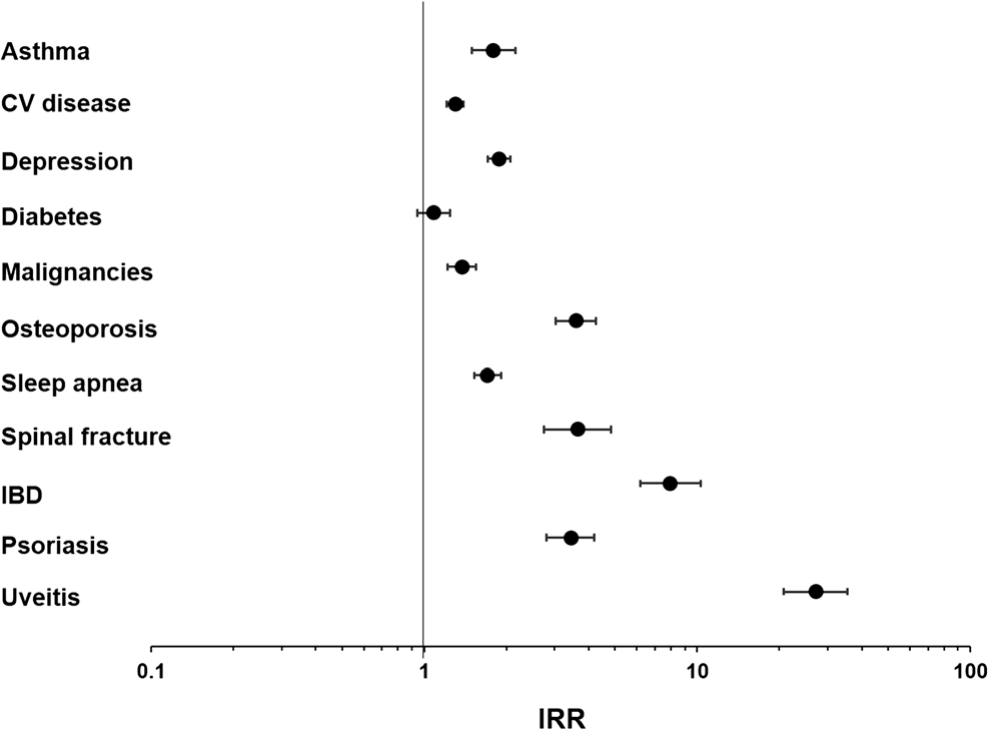
Incidence rate ratios of comorbidities in patients with *AS* vs their matched controls. *AS*, ankylosing spondylitis; *CV*, cardiovascular; *IBD*, inflammatory bowel disease; *IRR*, incident rate ratio

Except for diabetes, dyslipidemia, and Parkinson disease, patients with AS had significantly higher incidence rates of all other comorbidities compared with matched controls (Table 3 and Fig. 2). Comorbidities with the highest incidence rates were hypertension, dyslipidemia, and depression (patients with AS—12.98, 13.52, and 6.05 per 100 PYs, respectively; matched controls—10.33, 12.90, and 3.22 per 100 PYs, respectively). The greatest differences in incidence rates were observed in IBD and uveitis; the incidence rates for IBD and uveitis were 8 and 26 times higher, respectively, in patients with AS compared with matched controls. Patients with AS had three to four times higher incidence rates of multiple sclerosis, osteoporosis, and spinal fracture, and approximately two times higher incidence rates of asthma, depression, gastrointestinal ulcers, sleep apnea, and venous thromboembolism, compared with matched controls.

**Table 3:** Proportions of patients with new comorbidities and the incidence rates per 100 patient years

	Patients with AS *N* = 6679		Matched controls *N* = 19,951		*P* value
	*n* (%)	Incidence rate	*n* (%)	Incidence rate	
Comorbidities					
Asthma	178 (2.7)	1.37	302 (1.5)	0.76	< 0.001
Cardiovascular disease	1080 (16.2)	14.36	2795 (14.0)	11.06	< 0.001
Angina	104 (1.6)	0.79	240 (1.2)	0.60	0.027
Atherosclerosis	328 (4.9)	2.69	794 (4.0)	2.13	0.001
Cerebrovascular disease/stroke	185 (2.8)	1.41	442 (2.2)	1.12	0.010
Coronary artery disease	233 (3.5)	1.85	529 (2.7)	1.38	< 0.001
Hypertension	1044 (15.6)	12.98	2736 (13.7)	10.33	< 0.001
Myocardial infarction	118 (1.8)	0.89	284 (1.4)	0.71	0.046
Peripheral vascular disease	177 (2.7)	1.35	422 (2.1)	1.07	0.011
Venous thromboembolism	142 (2.1)	1.07	214 (1.1)	0.54	< 0.001
Depression	686 (10.3)	6.05	1187 (6.0)	3.22	< 0.001
Diabetes	273 (4.1)	2.30	752 (3.8)	2.13	0.242
Dyslipidemia	1208 (18.1)	13.52	3544 (17.8)	12.90	0.551
Gastrointestinal ulcers	93 (1.4)	0.70	156 (0.8)	0.39	< 0.001
Malignancies	394 (5.9)	3.24	879 (4.4)	2.35	< 0.001
Multiple sclerosis	19 (0.3)	0.14	16 (0.1)	0.04	< 0.001
Osteoporosis	286 (4.3)	2.26	250 (1.3)	0.63	< 0.001
Parkinson disease	14 (0.2)	0.10	32 (0.2)	0.08	0.402
Sleep apnea	445 (6.7)	3.76	827 (4.1)	2.21	< 0.001
Spinal fracture	107 (1.6)	0.81	89 (0.4)	0.22	< 0.001
Extra-articular manifestations of AS					
Inflammatory bowel disease	209 (3.1)	1.69	85 (0.4)	0.21	< 0.001
Crohn disease	127 (1.9)	0.99	40 (0.2)	0.10	< 0.001
Ulcerative colitis	136 (2.0)	1.05	59 (0.3)	0.15	< 0.001
Psoriasis	202 (3.0)	1.55	180 (0.9)	0.45	< 0.001
Uveitis	469 (7.0)	4.04	60 (0.3)	0.15	< 0.001

*AS*, ankylosing spondylitis

After controlling for patients’ demographic and baseline clinical characteristics, multivariate analyses showed that patients with AS had a significantly higher risk of developing asthma, cardiovascular disease, depression, hypertension, malignancies, osteoporosis, sleep apnea, spinal fracture, IBD (including both Crohn disease and ulcerative colitis) psoriasis, and uveitis compared with matched controls (Table 4). Increased risk of developing cardiovascular disease, depression, hypertension, osteoporosis, spinal fracture, IBD (including Crohn disease), and uveitis in patients with AS were consistent across different age groups (< 45 years, ≥ 45 to < 65 years, and ≥ 65 years). Patients with AS had an increased risk of developing sleep apnea compared with matched controls at all ages except < 45 years of age and had an increased risk of developing malignancies if they were < 65 years of age. Among patients with AS, women were shown to have an increased risk of developing asthma, depression, and osteoporosis compared with men; men were shown to have increased risk of developing cardiovascular disease, dyslipidemia, hypertension, malignancies, sleep apnea, and spinal fracture compared with women.

**Table 4:** Summary of Cox proportional hazard models for risk of developing a new comorbidity

	Hazard ratio (95% CI)^a^				
Newly diagnosed comorbidities	Patients with AS vs matched controls	Patients < 45 years: AS vs matched controls	Patients ≥ 45 to < 65 years: AS vs matched controls	Patients ≥ 65 years: AS vs matched controls	Male vs female patients with AS
Comorbidities					
Asthma	1.55** (1.21–1.98)	2.25** (1.43–3.54)	1.39 (0.99–1.96)	1.37 (0.80–2.33)	2.05* (1.50–2.79)
Cardiovascular disease^b^	1.41* (1.28–1.55)	1.55* (1.25–1.92)	1.36* (1.21–1.54)	1.50** (1.20–1.89)	0.88*** (0.77–1.00)
Depression	1.85* (1.64–2.09)	2.35* (1.89–2.91)	1.73* (1.47–2.05)	1.48*** (1.10–2.00)	1.95* (1.67–2.27)
Diabetes	1.02 (0.86–1.22)	0.94 (0.53–1.68)	1.07 (0.85–1.34)	0.95 (0.68–1.31)	1.10 (0.86–1.41)
Dyslipidemia	1.07 (0.98–1.17)	0.97 (0.76–1.24)	1.08 (0.97–1.21)	1.09 (0.90–1.31)	0.78* (0.69–0.88)
Hypertension	1.36* (1.25–1.50)	1.58** (1.25–1.99)	1.31* (1.16–1.47)	1.45** (1.18–1.78)	0.81*** (0.71–0.92)
Malignancies	1.39* (1.19–1.62)	1.76*** (1.06–2.91)	1.46** (1.18–1.79)	1.26 (0.98–1.63)	0.76*** (0.62–0.94)
Osteoporosis	3.77* (3.07–4.64)	26.37* (6.99–99.65)	4.11* (3.16–5.33)	2.64* (1.84–3.80)	1.82* (1.44–2.29)
Sleep apnea	1.55* (1.33–1.80)	1.39 (0.97–1.99)	1.62* (1.34–1.95)	1.53*** (1.01–2.32)	0.68** (0.56–0.84)
Spinal fracture	3.90* (2.79–5.45)	4.67*** (1.17–18.57)	4.29* (2.61–7.03)	3.40* (2.09–5.53)	0.58*** (0.38–0.89)
Conditions associated with AS					
Inflammatory bowel disease	6.66* (4.91–9.04)	9.65* (5.12–18.21)	6.38* (4.30–9.45)	4.36** (2.03–9.37)	1.18 (0.89–1.81)
Crohn disease	9.42* (6.20–14.30)	11.65* (5.44–24.98)	9.24* (5.30–16.10)	7.61** (2.63–21.99)	1.28 (0.90–1.82)
Ulcerative colitis	4.42* (2.93–6.67)	9.29* (3.78–22.84)	4.64* (2.77–7.77)	1.31 (0.37–4.63)	1.27 (0.89–1.81)
Psoriasis	2.18* (1.61–2.94)	3.00** (1.72–5.26)	2.12** (1.44–3.13)	1.38 (0.60–3.17)	0.98 (0.73–1.31)
Uveitis	22.73* (16.91–30.56)	25.86* (14.52–46.05)	25.09* (16.95–37.15)	11.99* (5.82–24.72)	0.79*** (0.65–0.96)

*AS*, ankylosing spondylitis

^a^Risk of newly diagnosed comorbidities for patients with AS relative to matched controls, adjusted for patient characteristics

^b^Cardiovascular comorbidities included angina, atherosclerosis, cerebrovascular disease, stroke, coronary artery disease, hypertension, myocardial infarction, peripheral vascular disease, and venous thromboembolism

*P < 0.0001

**P < 0.001

***P < 0.05

## Discussion

This study demonstrated that in this population of patients with either incident or prevalent AS, a greater proportion of patients with AS had baseline comorbidities compared with matched controls. In addition to a higher rate of extra-articular manifestations of AS (IBD, psoriasis, and uveitis), patients with AS also had significantly higher rates of asthma, cardiovascular disease (including angina, atherosclerosis, cerebrovascular disease/stroke, coronary artery disease, hypertension, myocardial infarction, and venous thromboembolism), depression, gastrointestinal ulcers, osteoporosis, sleep apnea, and spinal fracture. The higher baseline comorbidity burden in patients with AS than in the matched controls is consistent with results from prior studies, with the exception of diabetes, for which rates were found to be statistically similar between patients with AS and matched controls [12–21]. Although the reasons for this particular lack of association are unclear, the higher prevalence of diabetes in the USA (10.8% of population of ages 20 to 79) than some of the other countries where published AS studies were conducted (e.g., Sweden, 4.7%; China, 9.8%; Japan, 5.7%; Spain, 7.7%) may have obscured the difference in rates of diabetes between patients with AS and matched controls in this study [25]. While the association of AS with cardiovascular disease, IBD, depression, osteoporosis, and uveitis has been well studied, the association of AS with higher rates of sleep apnea, asthma, and malignancies is less established, and additional research is required to further characterize these comorbidities in AS populations.

Although the prevalence of psoriasis at baseline and the incidence of new cases of psoriasis were higher in patients with AS than in matched controls, the overall risk of newly diagnosed psoriasis was lower than expected. We suspect that, in this patient population, psoriasis was diagnosed before the diagnosis of AS, thus making it less likely to be identified as a new comorbidity.

In our study, patients with AS had an increased risk of developing asthma, which is consistent with results of a previous study showing an overall increased risk of asthma in patients with AS compared with the general population; our results are also consistent with the observation of a higher risk of asthma for women with AS than men with AS, with the highest risk being in patients 30 to 49 years of age [26]. This increased risk in younger adults observed in both studies may be due to older patients in general being more likely to already have asthma [27]. The higher rate of asthma in women with AS is likely related to the increased incidence of nonallergic asthma or adult-onset asthma in women overall [27, 28].

Patients with AS in our study were more likely than matched controls to develop malignancies. Previous studies have shown that patients with AS have an increased risk of developing cancer [18, 29]. In addition, a recent systematic review and meta-analysis showed an overall increased risk for malignancy in patients with AS; however, subgroup analyses did not confirm the association to be significant in American or European populations with an increased risk for malignancy of the digestive system, multiple myeloma, and lymphoma [30].

Previous studies have established that patients with AS have an increased risk of spinal fractures, and these fractures can be caused by minimal trauma [31–34]. Our study also showed that patients with AS had a higher risk of spinal fractures compared with matched controls, and the risk was higher in men with AS than women with AS. Remodeling, fusion, and osteoporosis likely contribute to the increased susceptibility to spinal fractures. Although women in our study had a higher rate of osteoporosis, a previous study has shown that men with AS have more severe radiographic damage than women with AS, which may contribute to the increased risk of spinal fracture in men vs women [35].

This study expanded on evidence from prior studies showing an increased incidence of sleep apnea in patients with AS [36–38]. Systemic inflammation has been theorized to contribute to the increased incidence of sleep apnea in patients with AS [36]. Tumor necrosis factor *α* levels are increased in sleep apnea independent of obesity [39]; however, the literature shows conflicting results on the effect of tumor necrosis factor inhibitors on sleep apnea [36, 40, 41]. In the current study, the risk of sleep apnea was significantly increased in patients with AS aged ≥ 45 years vs matched controls aged ≥ 45 years. Although the risk was also increased in patients with AS vs matched controls aged < 45 years, this difference was not significant.

These findings also support evidence from other published reports on the risk of developing new comorbidities in patients with AS, especially for cardiovascular diseases and depression [15, 17, 20, 42]. The results of this current study are consistent with those from the Swedish National Patient Register, which showed a 50% higher risk of acute coronary syndrome and vascular thromboembolism and a 25% higher risk of stroke in patients with AS compared with the general population [42]. In addition, a study using administrative claims data from the Taiwan National Health Insurance Database showed a > 2-fold increase in the risk of stroke in patients with AS vs a comparison cohort without AS [20]. The percentage of patients with AS newly diagnosed with depression in our study was similar to data from the Skåne Healthcare Register in Sweden, which showed an 80% increased rate of doctor-diagnosed depression in women with AS and a 50% increase in men with AS compared with age- and sex-specific rates of depression in the general population seeking care [17]. Studies of newly diagnosed osteoporosis in patients with AS are lacking, but a systematic literature review of the prevalence of low bone-mineral density found a high overall prevalence of decreased bone density (> 50%) and osteoporosis (13–16%) in patients with newly diagnosed AS (disease duration, < 10 years) [43]. In addition, a cross-sectional analysis of bone-mineral density measurements from 504 Chinese patients with AS showed a higher prevalence of osteoporosis (9.7 vs 0.0%) and osteopenia (57.5 vs 34.9%) compared with matched controls [44]. Although previous studies have shown the increased risk of comorbidities in patients with AS, all these studies were conducted outside of the USA. Our study provides new information and contributes to the existing knowledge about the increased risk of comorbidities in patients with AS and provides important real-world evidence from a US patient population.

This study did not examine the causality of the association between AS and comorbidities. A potential explanation is that patients have shared genetic or environmental risk factors that predispose them to both AS and to the comorbidities. Other possibilities are that AS may increase the risk of the evaluated comorbidities or that AS may be a marker for or consequence of unidentified risk factors that contribute to the risk of both AS and the comorbidities. The higher rates of baseline comorbidities in patients with AS may be evidence of interrelated disease processes or may be associated with treatment; however, associations with treatments were not tested as a part of this research.

The mean age of patients in the study was older than what has been reported in other studies of patients with AS. The patient population in this study included both incident and prevalent AS patients, and the older age may also be due to the inclusion criteria, which required continuous enrollment in an insurance plan for 12 months before and after the index date. Younger patients are more likely to switch jobs and subsequently switch insurance carriers more often [45]. In addition, younger patients may be less likely to have regular visits with healthcare providers, thus having fewer opportunities for a diagnosis of AS or a comorbidity.

## Limitations

The limitations of this study are those inherent to any retrospective database analysis. First, in the absence of patient charts or provider attestations, misclassification error is possible when relying on diagnosis coding from administrative claims data, where the extent of under- or overcoding for AS and comorbidities is unknown. In addition, because of the lack of data on body mass index, we were unable to evaluate obesity as a comorbidity or to control for obesity with related comorbidities such as diabetes, sleep apnea, and cardiovascular diseases. Further, we did not evaluate other risk factors that could contribute to the development of comorbidities (e.g., smoking, alcohol consumption, and use of other medications such as nonsteroidal anti-inflammatory drugs). Second, due to the large cohort sizes, small differences between comparator groups may be found to be statistically significant, even though the corresponding interpretation of clinical significance may be questionable. Third, patients with AS may visit healthcare providers more often than matched controls and thus may have more opportunities to be diagnosed with comorbidities. This potential difference in opportunities for diagnosis may result in overestimation of the difference in comorbidity rates between patients with AS and matched controls. Finally, the MarketScan administrative claims databases are convenience samples of individuals, employees, retirees, and their dependents in the USA who had commercial health insurance or Medicare supplemented by other health insurance paid for by a current or former employer; therefore, the results may not be applicable outside of this demographic.

## Conclusion

In this real-world, US claims-based study, patients with AS were shown to have significantly more comorbidities than matched controls. Understanding the frequency and risk for these diseases can assist providers with comorbidity screening and patient-management strategies. Furthermore, knowledge of comorbidity risks improves understanding of long-term health prognoses. Use of comprehensive longitudinal real-world data is a promising tool to evaluate the burden of comorbidities in patients with AS. Further research is needed to examine the potential cause and effect relationships between AS and comorbidities.
